# Quinine in Fevers

**Published:** 1849-10

**Authors:** Henry Hartshorne

**Affiliations:** Philadelphia


*Quinine in Fevers.* By HENRY HARTSHORNE, M. D., of Philadelphia.

An unpresuming statement of impressions formed recently, during a hospital residence, of the action of sulphate of quinine in autumnal fevers, has appeared to a southern correspondent of the Examiner, to have been "prematurely volunteered." Taking shelter under the modesty, evidently appropriate, with which these impressions were put forth, as mites in the balance of medical opinion, a few words more may be allowed upon the same subject.

It is asked, "what is *miasm-cause?*"—We rejoin, what is the cause of cholera?—what of the tubercular diathesis?—what of cancer—of yellow fever—of influenza? These questions are all upon a similar footing; yet does the critical writer deny the essential and practical entity of each or any of these? The peculiarities of the diseases termed miasmatic, are by all authority sufficient to determine their reference to a peculiar causation; our ignorance of the *nature* of which interferes no more with our knowledge of its existence, than would the uncertainty of the Pepsin theory with our trust in the digestibility of food.

The "Hospital Reports" in the American Journal of Medical Sciences, were written not to dare criticism, but to excite reflection; and accuracy of phrase has thus been sometimes sacrificed to brevity. Admitting, however, with this excuse, the guilt of making free with the word "antidote" with too little explanation of its intent, we would still reiterate for consideration the idea on which its use was based. As a matter of theory merely, it would be waste of words; but the subject has practical importance.

*Fever* is a symptom, or group of symptoms, the result of various pathological conditions. Venesection, purgation, and citrate of potash, &c., attack the symptoms directly, by their influence on the blood and circulatory system. If a large dose of arsenic excite fever by inflaming the intestines, the peroxide of iron may soon reduce, or might before have prevented that symptom, by addressing its cause. If an inflamed chancre should accelerate the pulse, its tranquilization by mercury might in part at least be ascribed to its anti-syphilitic power. If violent neuralgic pain, or mental inquietude, irritate the system into fever, large doses of narcotics or some moral agency may quell it, by addressing the condition from which it arose.

Is quinine, then, in large or small doses, a remedy which, like the lancet and the cathartic, and refrigerants, reduces symptomatic fever ? Or does it, like mercury in syphilis, or anodynes in neuralgia, attack a peculiar *condition* of the system, of which chills, fevers and sweats, are in turn the consequents ? If the latter question be answered in the affirmative, it is no very dangerous perversion of language to say that, besides being generally a tonic and nervous stimulant, the sulphate of quinine is an "antidote" for intermittents and remittents,—a class of diseases to which no less an authority than Dr. Wood (Practice, Vol. I. p. 229) has sanctioned the application of the term "miasmatic."

The *cause* we know not; the *condition* of system peculiar to these diseases (certainly the result of some cause) we cannot demonstrate with the scalpel; but we know it by its effects, its connection with localities, and relation to remedies.

If much larger doses of quinine are borne in the southern than in the northern states, is not this fact accompanied by the truth that in those states miasmatic diseases are much more violent, and hence their *cause* (whatever it be) may be inferred to be more intense and prevalent ? At least, this gives some approach to a reconciliation of the effects of the drug in these diseases with its generally understood physiological actions.

The matter may be summed up in a few queries, which it would be indeed premature for the present writer to attempt to answer. The experience of the north as well as the south must determine them.

Have our predecessors been in error, or not, in considering bark and its alkaline principles to be of the tonic class of remedies, and in prescribing them in all cases of prostration and debility ?

Is there any case of *purely inflammatory* disease, neither *endemic* nor epidemic, in which a full, hard, accelerated pulse, hot, dry skin, and intense headache, and thirst, will, under large doses of unadulterated quinine, become subdued, tranquilized, and entirely cured ?

Will a majority of cases of typhus and typhoid fever be relieved or aggravated by the administration of 30 grains of quinine once or twice daily, after the first day, and during the first week ?

If this discussion could lead to a clear decision on the subject by competent authority, our "impressions," however rashly formed, will not have been volunteered in vain.

